# An Adaptive Framework for Real-Time ECG Transmission in Mobile Environments

**DOI:** 10.1155/2014/678309

**Published:** 2014-07-03

**Authors:** Kyungtae Kang

**Affiliations:** Department of Computer Science and Engineering, Hanyang University, Ansan 426-791, Republic of Korea

## Abstract

Wireless electrocardiogram (ECG) monitoring involves the measurement of ECG signals and their timely transmission over wireless networks to remote healthcare professionals. However, fluctuations in wireless channel conditions pose quality-of-service challenges for real-time ECG monitoring services in a mobile environment. We present an adaptive framework for layered coding and transmission of ECG data that can cope with a time-varying wireless channel. The ECG is segmented into layers with differing importance with respect to the quality of the reconstructed signal. According to this observation, we have devised a simple and efficient real-time scheduling algorithm based on the earliest deadline first (EDF) policy, which decides the order of transmitting or retransmitting packets that contain ECG data at any given time for the delivery of scalable ECG data over a lossy channel. The algorithm takes into account the differing priorities of packets in each layer, which prevents the perceived quality of the reconstructed ECG signal from degrading abruptly as channel conditions worsen, while using the available bandwidth efficiently. Extensive simulations demonstrate this improvement in perceived quality.

## 1. Introduction

Millions of people die each year from coronary heart disease; the elderly are more susceptible to such diseases. Many retirement homes are installing systems that can continuously and wirelessly monitor the electrocardiograms (ECGs) of some of their residents. For example, AlarmNet [1] is an assisted-living and residential monitoring network that opens up new opportunities for continuous monitoring of the elderly and those in need of medical assistance. Wearable ECG sensors can wirelessly monitor a patient's heartbeat, alerting healthcare personnel to changes in status while simultaneously delivering data to a back-end archival system for longer-term storage [2].

However, to make ECG monitoring successful in retirement homes, we need to be able to monitor several patients in real time over a limited-bandwidth wireless connection. This is a challenging task for the following reasons.

*(i) Large Volume of Data*. The amount of uncompressed data is large, even for a single patient. A day's worth of data from a 12-lead ECG with 11-bit resolution and a sampling frequency of 300 Hz will occupy around 500 MB. Transmitting these amounts of data over a wireless link in real time is challenging because of the following.

*(ii) Bandwidth Fluctuations*. The throughput of a wireless channel may be reduced because of multipath fading, cochannel interference, and noise; the capacity of a channel is likely to depend on the distance between the base station (BS) and the wireless transmitter, which will change as a patient moves about. Moreover, when a patient's device switches between cells, the available bandwidth may change drastically: in the worst case, a BS may not have enough capacity remaining to accept a new connection.

*(iii) High Channel Error Rate*. Wireless channels are typically much noisier than wired links and suffer from both multipath fading and shadowing, which can have a devastating effect on the perceived quality of a reconstructed ECG signal.

Although several researchers have reported success in the remote ECG monitoring of patients [3–5], the constraints of wireless transmission have not been addressed. These projects involved the real-time acquisition of ECG data and its transmission over the Internet (wired and wireless) without considering bandwidth constraints and the effects of transmission errors on the ECG signal. Most of these studies involved a single subject monitored with few electrodes, thus generating limited data: some [6–8] focused on short-range transmission of ECG data; others [9] considered wireless connectivity in the remote monitoring of cardiac activity. Various compression techniques [10–16] can reduce the amount of data and hence the transmission time. However, compression alone is not sufficient to cope with a congested wireless channel that has high PER.

In this paper, we analyze the errors introduced into ECG data during transmission, using both quantitative and qualitative criteria, and then present an adaptive framework for high-quality ECG communication over wireless networks. This framework consists of a layered coding for ECG data and a cooperating ARQ- (Automatic Repeat request-) based error control scheme, combined with a layer-based EDF (LB-EDF) scheduler to support the scalable and reliable monitoring of remote patients. In this scalable representation, a group of samples is split into a base layer (BL), which contains sufficient details to allow serious cardiac abnormalities to be observed, and several enhancement layers (ELs), which increase the amount of details visible in the reconstructed ECG signal. This layered representation also serves to interleave a packet containing erroneous data with others, so that it is distributed across the time domains. This reduces the influence of packet errors, which frequently occur in bursts during transmission over a wireless channel, on the readability of the ECG signal received by the remote monitoring station (RMS).

In combination with this representation scheme, the LB-EDF scheduling algorithm is designed for effective delivery of scalable ECG data over a lossy channel in real time. Scalable ECG data has timing constraints because of its sensitivity to delay and jitter. The EDF policy discards the higher and less important ELs, if necessary, to give BL and the lower ELs a greater chance of arriving at the RMS on time. Together with the ARQ scheme, the LB-EDF scheduler greatly improves the readability of the ECG signal at the RMS.

Our adaptive framework has the following key features.

*(i) Graceful Quality Degradation*. The layered ECG coding adapts to bandwidth variations and less important packets can be successively dropped as channel conditions worsen, so that perceived quality degrades gracefully. 

*(ii) Efficiency*. The available bandwidth is used efficiently in a way that maximizes the perceptual quality and the resulting ECG readability, thus facilitating a correct diagnosis.

In this paper, we are proposing an infrastructure for wireless monitoring of mobile patients, like those put forward by Varshney [17, 18], Baker and Hoglund [19], Malan et al. [20], and Kang et al. [21], among others. These authors review the ways in which a wireless infrastructure forms the basis for reliable systems for pervasive healthcare. However, none of these papers consider the use of scalability to respond to bandwidth constraints and the packet errors that occur in mobile environments.

The rest of this paper is organized as follows. In Section 2, we introduce the service architecture we adopted in this study for remote ECG monitoring. In Section 3, we propose scalable ECG coding that allows an effective response to bandwidth fluctuations and can tolerate the error rates of wireless channels. In Section 4, we present our link-level error control scheme and the associated LB-EDF scheduling policy. In Section 5, we introduce our evaluation environment and investigate the effectiveness of the adaptive framework that we are proposing. In Section 6, we draw conclusions.

## 2. System Architecture

### 2.1. Wireless System Architecture for Remote ECG Monitoring

**Figure 1:**
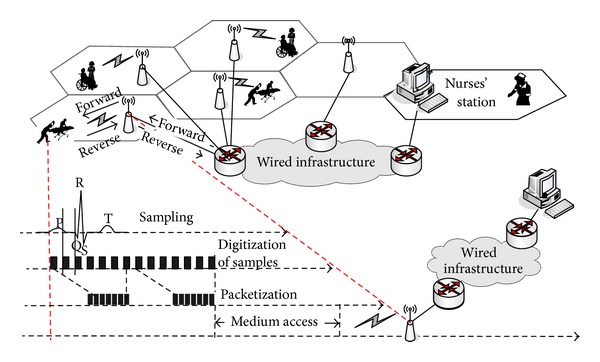
Wireless system architecture.

Figure 1 shows the architecture of the proposed system for remote ECG monitoring. A wearable wireless ECG sensor that consists of multiple electrodes, a wireless transmitter, and a receiver are used to continuously measure the heart activity of a mobile patient. The resulting digital stream is grouped into packets that then transmitted wirelessly to remote healthcare professionals in real time over a secure wireless connection such as WLAN, GSM, or CDMA.

In the proposed wireless system, the ECG monitoring application is developed based on the infrastructure mode, which requires a fixed base station (BS) to be connected to an established fixed network infrastructure. This BS provides a communication portal for wireless ECG sensors worn by patients within its range. The data collected from the ECG sensors worn by multiple patients is transmitted wirelessly to a nearby BS, following which the BS relays this data over wired infrastructure to an RMS, as shown in Figure 1. Wired infrastructure transmits data at higher speeds and with greater reliability than a wireless network, and therefore, all data losses and transmission delays can be attributed to the wireless network without significant inaccuracy. Therefore, in this study, we ignore details pertaining to transmission through the wired infrastructure, and we assume that the ECG sensor and the RMS are connected through a single wireless link for simplicity.

### 2.2. Multiple Access of Wireless Physical Channel

One of the major issues based on ECG monitoring using wireless infrastructure is the reliable and predictable delivery of patient cardiac information. In the aforementioned system architecture, medical devices worn by multiple patients attempt to transmit collected clinical data to the corresponding remote professionals via a wireless channel. Therefore, appropriate channel access control is required to effectively coordinate their access, while ensuring timely and predictable delivery of real-time medical data [22].

For this purpose, we assume that channel access to an appropriate BS by medical devices worn by patients is compatible with code division multiple access (CDMA), with a slot structure in which each slot lasts for a particular period. Multiplexing techniques such as CDMA are used in conjunction with time division multiple access (TDMA) to maximize both the individual user's throughput and the overall system throughput. This form of channel access can help ensure the predictable delivery of clinical data by assigning dedicated code to the transmitter of each medical device. In addition, TDMA allows multiple applications running on a medical device to send or receive data through a wireless channel in a predictable manner by assigning dedicated time-slots to each application. It should be noted that this way of channel access can also be supported in IEEE 802.11 wireless networks by effectively exploiting the point coordination function (PCF) [23, 24], which has been proposed in the original IEEE 802.11 standard as an optional access mechanism. PCF implements a centralized polling scheme to support synchronous data transmissions, where the point coordinator performs the role of a polling master.

If each time-slot lasts *τ* seconds and if the size of a packet (in this paper, “packet” refers to a* physical-layer packet* unless specified otherwise) is *L*_pkt_ bits, the channel data-rate *μ* is given by *L*_pkt_/*τ* bits per second, assuming that the transmission of a packet requires one time-slot. For example, the fundamental timing unit for packet transmission in a CDMA2000 1xEV-DO Revision A [25] system is 1.67 ms, and the reverse data channel of this system supports data-rates in the range of 4.8 kb/s to 1843.2 kb/s, depending on the packet size, modulation, and coding. Based on periodic feedback, regarding the channel state of a mobile device, obtained through the time division multiplexed data-rate control (DRC) channel, the BS adaptively controls the transmission rates of the device.

**Figure 2:**
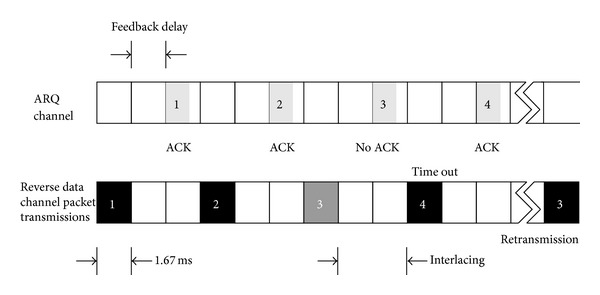
Timing of periodic packet transmission or retransmission over reverse data channel.

However, in this study, we assumed that the length of a packet is set to *L*_pkt_ bits, with the length of each packet payload being *L*_payload_ bits. In addition, the transmission of each packet over the reverse data channel is assumed to require a single time-slot that has duration of *τ* seconds, whereas successive packets are periodically interlaced with *φ* time-slots, as shown in Figure 2. When the channel data-rate of the reverse channel is set to *μ* bits per second, the effective channel data-rate available for an ECG application is *μ*_*e*_ = *μ*/(*φ* + 1) bits per second. The corresponding data-rate of the payload dedicated to the ECG application is *μ*_*e*_(*L*_payload_/*L*_pkt_) bits per second, which must be higher than that required by the application.

To reduce the PER in the upper layers and thus to provide reliable ECG monitoring, we introduce a MAC-layer ARQ mechanism with quick turnaround time to recover packet errors. The ARQ channel, which is carried over the forward MAC channel as shown in Figure 2, enables the BS to transmit acknowledgement (ACK) or negative ACK (NAK) responses to the medical sensor after *δ*-slot delay. Then, the MAC-layer payload contained in a packet that failed to be delivered is rescheduled for transmission. The proposed scheme for the scheduling of packet transmission at the medical sensor is explained later.

## 3. Representation of ECG Data

To enable reliable remote monitoring, ECG data must be delivered within a reasonable time with minimal corruption. However, the wireless channels between the ECG sensors worn by patients and the BSs are typically very noisy and experience multipath fading and shadowing; this increases the PER considerably when packet errors occur in clusters with relatively long error-free intervals between them. Furthermore, the PER varies significantly as channel conditions change over time with changes in the wireless environment, especially when a patient moves. This leads to fluctuation in the throughput of a wireless channel. In such situations, it is necessary to prevent a significant decrease of signal presentation in order to reduce the risk of misinterpretation by remote healthcare professionals; therefore there is a critical need for an efficient scheme for the representation of ECG data and the robust transmission of ECG data over wireless channels. In this section, we present an appropriate scheme for the representation of ECG data, following which we introduce a link-level error control scheme for the robust transmission of ECG data over wireless channels in the next section.

In general [26], an *η*-lead ECG is one in which *η* different electrical signals are recorded almost simultaneously, and it is often used as a one-off recording of an ECG. If *η* leads are recorded and if the ECG output for each lead is digitized at a rate of *R* samples per second, each of which has a resolution of *L*_smpl_ bits, the resulting data-rate *μ*_ecg_ of the wireless ECG application is given as

(1) $$\begin{array}{c} \mu_{\text{ecg}}=\eta RL_{\text{smpl}}. \end{array}$$

The digital stream is packetized and then sent to a remote monitoring device over a wireless channel.

**Figure 3:**
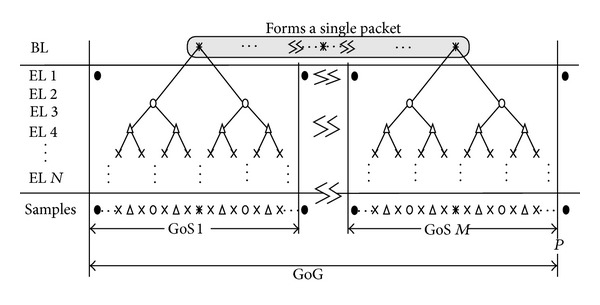
Framework for layered temporal scalability and packetization for transmission over the wireless channel.

It is clear from this definition that the quality of the obtained ECG signal improves with an increase in the sampling frequency or resolution of each sample. This scalability plays a crucial role in delivering the best possible service quality over unpredictable wireless networks, and it enables an application to adapt the quality of the streamed ECG service to dynamically changing network conditions. In a standard environment, scalability is achieved through a layered structure, where the ECG information is divided into two or more discrete bit streams corresponding to different layers, as shown in Figure 3. The base layer (BL) ECG stream contains fundamental ECG information that is periodically sampled at a low frequency. The enhancement layer(s) (EL(s)) contains ECG data sampled at higher frequencies in different time domains to produce the expected scalability; when combined with the BL-stream, it progressively delivers higher data-rate and a more sophisticated signal quality.

Temporal scalability involves the partitioning of a group of samples (GoS) into a single BL and multiple ELs. Samples at the center of each GoS are packed into a single BL packet according to their sequence numbers; then this BL packet is transmitted with the highest priority. Assuming that the size of a packet and that of each ECG sample is *L*_payload_ and *L*_smpl_, respectively, constructing a single BL packet requires *M* = *L*_payload_/*L*_smpl_ GoSs, and the interval of *M* GoSs is known as the period *P*. Packet transmission from the ECG sensor begins after all the samples of the first *M* GoSs are generated, buffered, and packetized. A layered structure for representing ECG data with *N* ELs is shown in Figure 3.

Now, let *S*_0_ and *S*_*n*_  (1 ≤ *n* ≤ *N*) be a set that contains ECG samples corresponding to the BL and the *n*th EL, respectively; *σ*_*i*,*j*_ is the *i*th ECG sample of the *j*th GoS. Then, *S*_1_ includes the first ECG sample from each GoS; thus, it is defined as

(2) $$\begin{array}{c} S_{1}=\left\{\sigma_{1,j}\;|\;j=1,2,3\ldots \right\}. \end{array}$$

The BL sample in the *j*th GoS is located at (*σ*_1,*j*_ + *σ*_1,*j*+1_)/2. Next, the set *S*_2_ contains two elements from each GoS, and it can be defined as follows:

(3) $$\begin{array}{c} S_{2}=\left\{\frac{\sigma_{1,j}+\left(\sigma_{1,j}+\sigma_{1,j+1}\right)/2}{2},\frac{\sigma_{1,j+1}+\left(\sigma_{1,j}+\sigma_{1,j+1}\right)/2}{2}\right\}. \end{array}$$

The first element of the set *S*_*n*_ (*n* ≥ 2) corresponds to the (2^(*N*−*n*)^+1)th ECG sample, whereas the interval between two consecutive elements of *S*_*n*_ is 2^*N*−*n*+1^. Therefore, set *S*_*n*_ can generally be defined as follows:

(4) $$\begin{array}{c} S_{n}=\left\{\sigma_{2^{N-n}+1,1}+2^{N-n+1}k\;|\;k=1,2,3\ldots \right\}. \end{array}$$

The number of samples in the BL |*S*_0_^*g*^| and in the *n*th EL |*S*_*n*_^*g*^|  (*n* ≥ 1) in a period *P* is, respectively, defined as follows:

(5) $$\begin{array}{c} \left|S_{0}^{g}\right|=M,\;\;\left|S_{n}^{g}\right|=2^{n-1}M\;\left(n\geq 1\right). \end{array}$$

Now, the total number of samples *R*_*g*_ in a period *P* is

(6) $$\begin{array}{c} R_{g}=M\overset{N}{\underset{n=0}{\sum }}\left|S_{n}^{g}\right|=2^{N}M, \end{array}$$

and the period *P* can be derived as follows:

(7) $$\begin{array}{c} P=\frac{R_{g}}{R}. \end{array}$$

As a result, the sampling frequency in the BS and in the *n*th EL is *R*_*B*_ = *M*/*P* and *R*_*E*_^*n*^ = 2^*n*−1^ *M*/*P*, respectively, where *R* = *R*_*B*_ + ∑_*n*=1_^*N*^ *R*_*E*_^*n*^.

**Figure 4:**
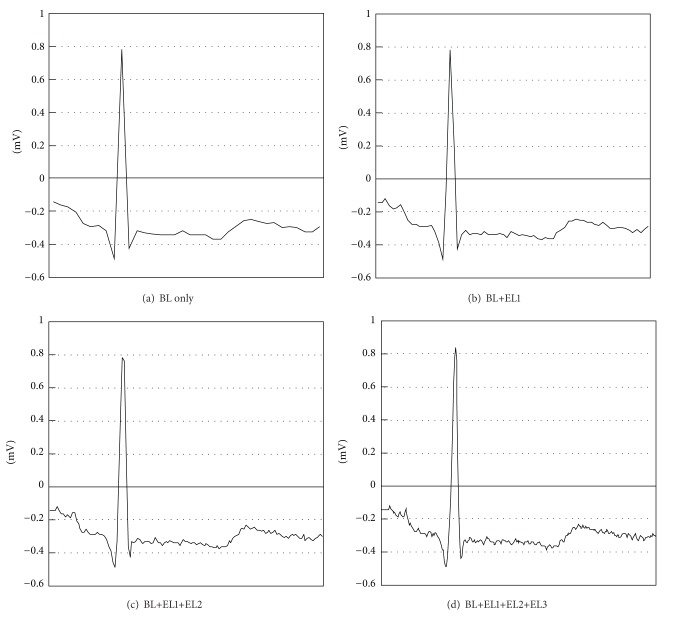
Temporal scalability of ECG signal for lead II, which is the voltage between the (positive) left leg electrode and the right arm electrode, as the number of ELs increases, for the duration of one second. The maximum sampling frequency is 360 Hz; thus, the maximum number of available ELs is 3 (*N* = 3).

Using the layered representation introduced above, Figure 4 shows that the quality of the ECG signal improves as more EL data are appended to the BL data, as expected. Further fine-grained scalability can be accomplished by increasing the maximum sampling frequency and the corresponding number of available ELs.

The proposed layered representation ensures that all the sample errors in an erroneous packet are interleaved across different time domains. As a result, it reduces the influence of packet errors, which frequently occur during transmission over a wireless channel, on the resulting readability of the ECG signal received by the RMS. Additionally, the proposed layered representation in conjunction with priority-driven link-level error control can effectively limit the effect of error bursts that are commonly occur in a wireless channel, especially when a patient moves around; this ensures that the* perceptual quality degrades gracefully under severe channel conditions*.

## 4. Link-Level Error Control

### 4.1. ARQ-Based Error Control

**Figure 5:**
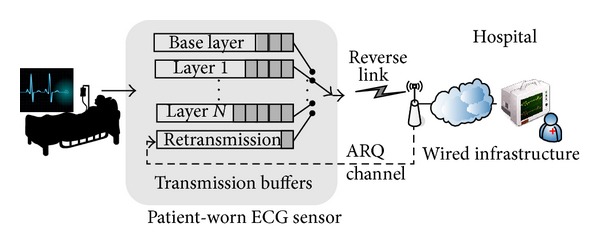
Structure for packet transmission and retransmission over wireless channel.

To enable high-quality ECG monitoring over a wireless channel, link-level error control is required. As mentioned in Section 2.2, we adopt an ARQ-based retransmission scheme to enable reliable data transmission over a wireless channel.  Figure 5 shows the error control structure for reliable ECG streaming. The buffers in the ECG sensor are used to provide some tolerance for variations in the network delay and the data consumption rates. The scheduler in the ECG sensor controls the packet transmission sequence.

The ECG streaming sequence consists of many packets, which are partitioned into a BL or several ELs. These packets are temporarily stored in the ECG sensor's transmission buffer, where they remain until they are scheduled for transmission. Some packets may be lost or damaged during transmission; such packets are reported by the BS to the ECG sensor via a forward ARQ channel, and they are stored in the buffer for retransmission. The scheduler of the ECG sensor selects one packet at a time from the buffer and transmits it over the lossy channel.

Now, it is important to select and schedule the packet delivery of ECG streaming data over a lossy network. Owing to the fixed delay constraint, not all lost packets can be recovered by retransmission. However, if the ECG sensor schedules a packet for transmission well before its display time, this packet will have a greater opportunity for retransmission before it is too late for display. If a packet is not available at its expected display time at the RMS, it will miss its deadline. Our objective is to develop a packet transmission policy that can select packets to be transmitted or retransmitted at any given time during an ECG streaming session in such a manner that the perceived quality of the signal reconstructed at the RMS is improved.

### 4.2. Problem Formulation

Let *p*_*α*,*n*_ denote the *α*th packet of the *n*th EL. The packets are stored in the transmission buffers once they are constructed. The release-time *r*_*α*,*n*_ is the time at which packet *p*_*α*,*n*_ becomes ready for scheduling in the transmission buffer. Let *t*_1_ be the time when the first ECG sample of a patient is obtained; then, the release-time can be calculated as follows:

(8) rα,n={t1+M+N−nREn+M−1REn(α=1),t1+M+N−nREn+M−1REn+(α−1)MREn(α>1).

Deadline *d*_*α*,*n*_ is the time by which packet *p*_*α*,*n*_ should be sent to the BS; otherwise, it is too late for display. Therefore, the deadline can also be defined as the time for which the first sample of packet *p*_*α*,*n*_ must be sent, which is the earliest time among the times required by the samples in that packet. Assuming an initial buffering time of one period, deadline *d*_*α*,*n*_ can be defined as follows:

(9) dα,n={t1+P+N−n+1REn+N−n+1REn(α=1),t1+P+N−n+1REn+(α−1)M−1REn(α>1).

Schedule time *s*_*α*,*n*_ is the time at which the scheduler sends packet *p*_*α*,*n*_ to the BS. The round-trip time (RTT) is defined as the interval from the time at which a packet is sent from the ECG sensor to the time at which the sensor receives feedback regarding this packet from the BS. The transmission time of packet *p*_*α*,*n*_ over the wireless channel is *c*_*α*,*n*_ = *L*_pkt_/*μ* = *τ*; thus, the RTT is 2*c*_*α*,*n*_. The time required by packet *p*_*α*,*n*_ to arrive at the BS is *f*_*α*,*n*_ = *s*_*α*,*n*_ + *c*_*α*,*n*_.

Packet *p*_*α*,*n*_ can be ready for scheduling if its arrival time is earlier than its deadline; that is, *t*_*c*_ < *d*_*α*,*n*_. A real-time scheduling algorithm for the delivery of scalable ECG streaming data over a lossy network is presented in the following section.

### 4.3. LB-EDF Scheduling Algorithm

Owing to the proposed layered representation of ECG data, it is intuitive to consider relative “importance” of the data in the scalable ECG stream in order to avoid abrupt degradation in the quality of the ECG signal. The loss of consecutive ECG symbols has a greater effect on the ECG signal than the loss of a few random symbols. Therefore, it is desirable to prioritize the delivery of packets in the BL or lower ELs, even under severe channel conditions. For this purpose, we assign higher priority to packets in the lower layer; these can then be transmitted earlier, with a greater opportunity for retransmission in the case of loss. Packets in the same layer are served according to earliest deadline first (EDF) policy. The scheme improves bandwidth utilization and the readability of the ECG signal in the case of some data loss via the prioritization of the low-frequency data in the BL or lower ELs. A detailed description of the algorithm is provided in Algorithm 1.

## 5. Performance of Wireless ECG Transmission

### 5.1. Simulation Method

#### 5.1.1. Statistics of Packet Errors in Wireless Channels

Zorzi et al. [27, 28] considered a binary process that describes the success or failure of packet transmissions for a simple* threshold model* of a channel in which a packet is successfully received if and only if the value of the fading envelope exceeds a certain threshold. It has been shown that such a process can be adequately approximated by a binary Markov process, that can be specified by two independent parameters *q* and *r*; *q* is the probability that the transmission of the *i*th packet is unsuccessful given that the (*i* − 1)th packet is transmitted successfully and *r* is the probability that the transmission of the *i*th packet is successful given that the (*i* − 1)th packet is not transmitted successfully. The Markov parameter *r* of the threshold model can be expressed as follows:

(10) $$\begin{array}{c} r=\frac{Q\left(\theta ,\rho \theta \right)-Q\left(\rho \theta ,\theta \right)}{e^{-\log(1-\epsilon )}-1},\;\text{where}\;\;\theta =\sqrt{\frac{-2\log\left(1-\epsilon \right)}{1-\rho^{2}}}, \end{array}$$

where *ϵ*  ( = *q*/(*q* + *r*)) is the steady-state PER and *Q*(·, ·) is the Marcum-*Q* [29] function. The term *ρ* is the correlation coefficient of two samples of the complex Gaussian fading process and is equal to *J*_0_(2*πf*_*D*_ *L*_phy_/*μ*), where *J*_0_(·) is the Bessel function of the first kind and of the zeroth order and *f*_*D*_ is the Doppler frequency. Note that the mobile speed is given by *f*_*D*_ *c*/*f*_*c*_ [30], where *c* is the speed of an electromagnetic wave and *f*_*c*_ is the carrier frequency.

#### 5.1.2. Simulation Setup

**Figure 6:**
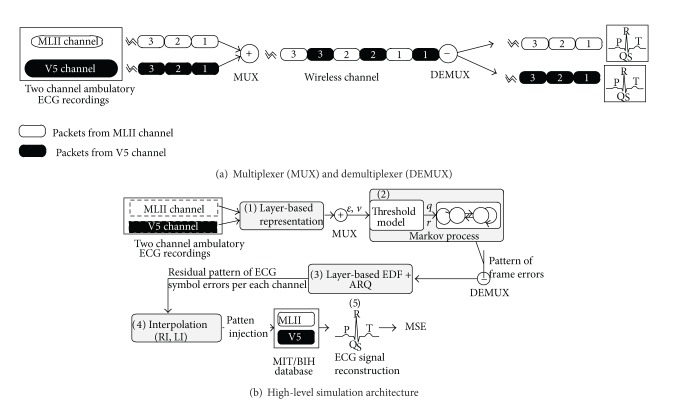
Simulation structure for QoS evaluation.

We used the MIT-BIH [31] arrhythmia database, which contains two-channel ambulatory ECG recordings obtained from 47 subjects studied by the BIH Arrhythmia Laboratory. The recordings were digitized at 360 samples per second for each channel, with 11-bit resolution; thus, the data-rate *μ*_*s*_ of each channel is 3960 b/s. Because we used two-channel ambulatory ECG recordings, data packets from both channels are multiplexed alternately before transmission, as shown in Figure 6(a), and the resulting total data-rate *μ*_ecg_ from the ECG sensor is 7920 b/s. We selected the data-stream 100.dat from the database. In this record, the upper signal is a modified limb II (MLII) lead and the lower signal is a modified V5 lead, which is one of the precordial leads.

First of all, ECG symbols generated from each channel are packetized using the layered representation we proposed in Section 3 ((1) in Figure 6(b)). The behavior of packet errors which arise in data transmission over the wireless channel was obtained by simulating the binary Markov process we described in Section 5.1.1 ((2) in Figure 6(b)). The corrupted packets are then scheduled to be retransmitted using the proposed LB-EDF algorithm ((3) in Figure 6(b)). All these processes were implemented in C and compiled and run on Linux.

**Figure 7:**
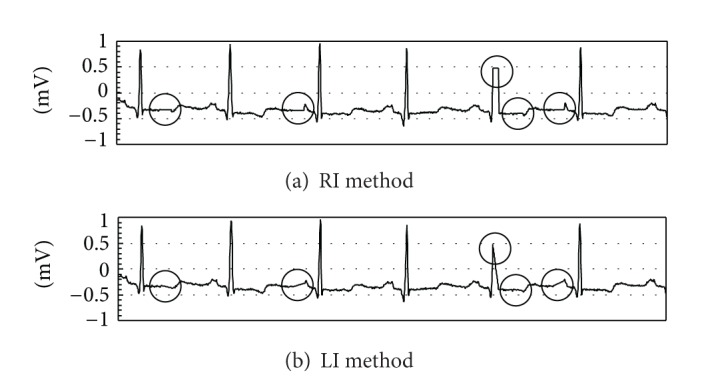
Interpolation of lost samples.

Packets that still contain errors after retransmission or miss their deadlines have to be discarded, and the samples that they contain are lost. To avoid a gap in the reconstructed waveform, we can replace the missing samples by interpolation ((4) in Figure 6(b)), which is conveniently performed in the RMSs in the application layer of the protocol stack. We considered two methods of interpolation, as shown in Figure 7.

*(i) Refresh Interpolation (RI)*. The value of the last sample transmitted successfully successively replaces the value of all the erroneous samples.

*(ii) Linear Interpolation (LI)*. The missing samples are replaced by values lying on the straight line that connects the values of previous and next samples transmitted successfully.

The interpolation methods that we propose do not insert new values for symbols that were not present in the original signal. The default values that are substituted for samples lost during transmission are only used to calculate mean-squared error (MSE) values that relate the original samples to the received samples. The proposed interpolation methods do not significantly change the low-frequency pattern of the ECG signal and will not affect interpretation of these signals by medical staff.

Finally, the ECG signals are reconstructed and evaluated by determining the MSEs, which quantify the difference between the reconstructed ECG signals, which have errors and the original signals retrieved from the database. The MSE for the time interval *t* can be estimated as follows:

(11) $$\begin{array}{c} \text{MS}{\text{E}}_{t}=\frac{1}{\eta t}\overset{N_{s}t}{\underset{i=1}{\sum }}{\left(s_{i}-{\hat{s}}_{i}\right)}^{2}, \end{array}$$

where *s*_*i*_ corresponds to the digitized value of the *i*th ECG signal obtained from the patient and ${\hat{s}}_{i}$ corresponds to the digitized value of the *i*th ECG signal reconstructed at the RMS after wireless transmission.

#### 5.1.3. Simulation Parameters

In the simulation, we set the packet size to 512 bits; each packet contains a maximum payload of 490 bits and a packet header of 22 bits. The fundamental timing unit for packet transmission is set to 1.67 ms, as per the CDMA2000 1xEV-DO Revision A standard. The transmission of a packet requires one time-slot, and the resulting reference channel data-rate is 307.2 kb/s.

Because patients at a hospital can be expected to have low mobility, we assumed a speed *v* in the range of 2 km/h to 5 km/h. We also varied the steady-state PER *ϵ* of the ECG sensors attached to a patient between 0 and 0.1. Then, we analyzed the service reliability of an ECG application using the proposed error control architecture for different interpolation methods. All the parameters used in our simulation are listed in Table 1, and the values of the Markov parameters corresponding to the sample PERs and mobile speeds are listed in Table 2.

### 5.2. Evaluation Results

**Figure 8:**
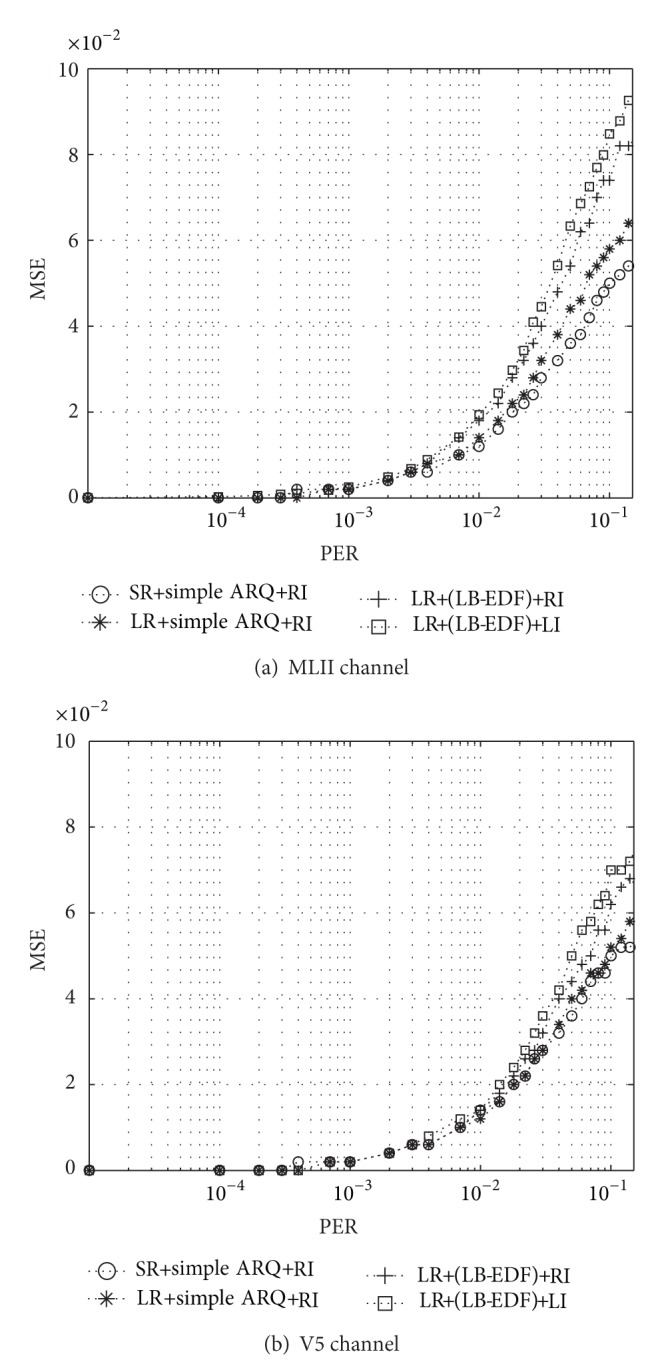
Results for MSEs when a patient moves at around 2 km/h.

**Figure 9:**
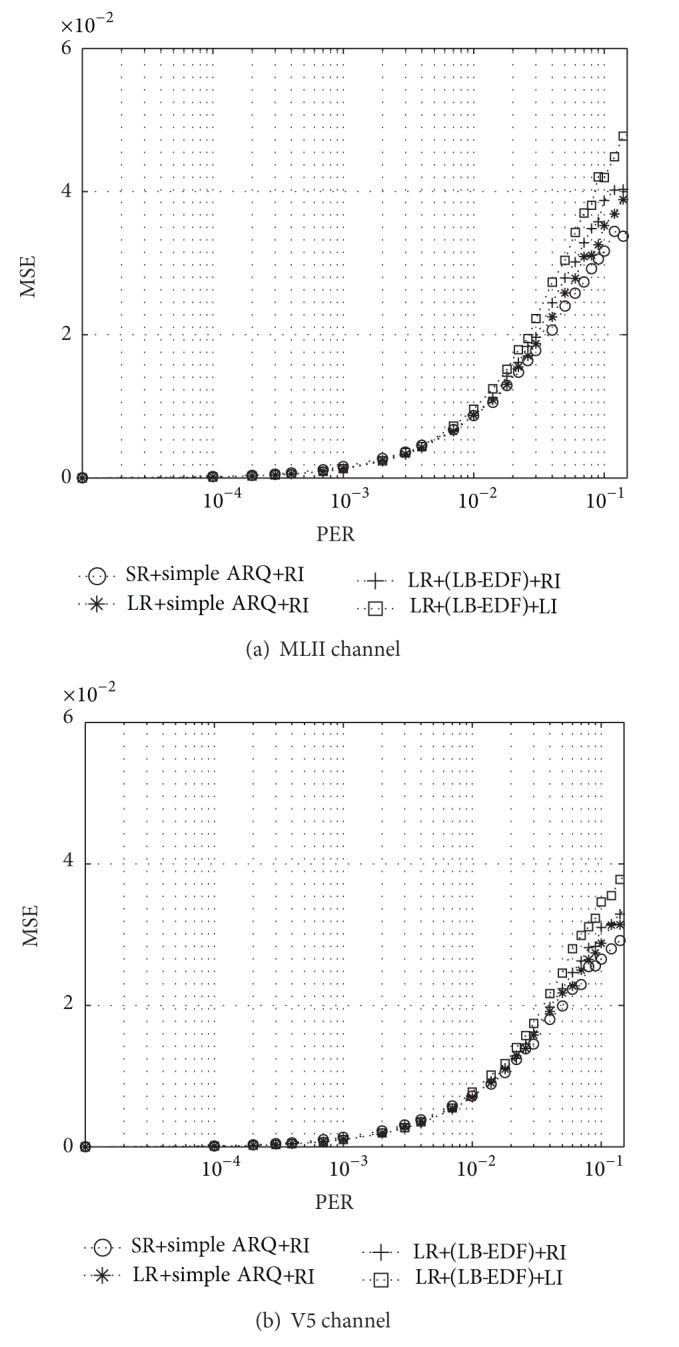
Results for MSEs when a patient moves at around 5 km/h.

Figures 8 and 9 show the performance of our framework in comparison with that of the conventional transmission framework (CTF), which serially packetizes consecutive symbols in order (which is denoted by serial representation (SR) in the figures) and employs first-in-first-out (FIFO) scheduler with priority-unaware error control based on ARQ. We also applied the RI method to the CTF to enable its direct comparison with our framework. The results are shown in terms of MSE for PER values ranging from 0 to 0.1. In general, MSE is relatively high in the case of slow-moving patients, where packet errors tend to occur in bursts separated by relatively long error-free intervals. This has an unfavorable effect on error recovery. It is also plausible that the MSE value increases with the PER.

We can clearly see three major trends from these in these two figures. First of all, there is an improvement in MSE even when the ECG data is transmitted using only the proposed layered representation. This is because the proposed representation interleaves consecutive symbols across different packets, making the system more robust to packet errors. However, the relative advantage decreases with the increase in the tendency of packet errors to occur in bursts, as shown in Figures 8(a) and 9(a). A similar phenomenon can also be found in the case of the ECG signal obtained from the V5 channel, as shown in Figures 8(b) and 9(b). Second, when ARQ and the LB-EDF scheduler are applied synergically together with the layered representation, the MSEs drop regardless of the interpolation schemes integrated; however, the relative advantage reduces slightly as the speed of mobility decreases. Finally, both interpolation schemes help reduce MSE and increase the readability of the reconstructed signals. However, the LI scheme achieves smoother interpolation in most cases. All these trends imply an overall improvement in the quality of ECG monitoring and the accurate diagnosis of cardiac status.

**Figure 10:**
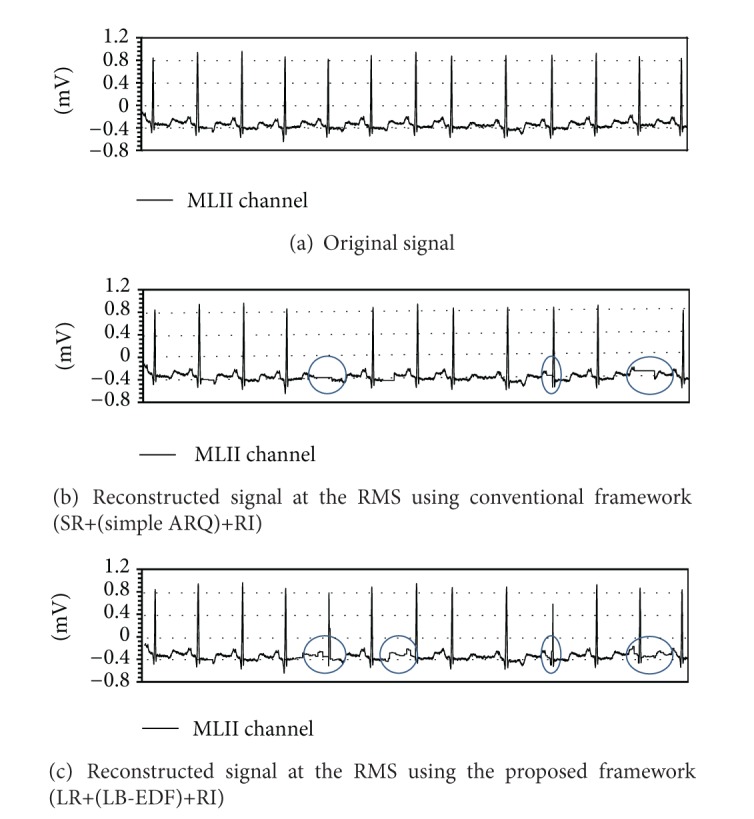
Snapshot of ECG signal fluctuations for MLII channel when PER is 0.1 and the patient moves at 2 km/h.

The relative advantage of our framework can clearly be seen in Figure 10, which depicts a snapshot of the original ECG signal obtained from patient and that of the corresponding signal reconstructed in the RMS under severe channel conditions (*ϵ* = 0.1 and *v* = 2 km/h). In such a situation, many residual erroneous packets often remain even after error control, both in our framework and the CTF. It is observed that compared to the original ECG signal in Figure 10(a), the ECG signal reconstructed with CTF frequently omits important ECG information; this might lead a physician to misinterpret a patient's condition. For example, the original ECG signal has 13 spikes, whereas the ECG signal reconstructed with CTF has only 11 spikes, as shown in Figure 10(b). As a result, in spite of the fact that the original ECG diagnosis is normal sinus rhythm with an atrial premature beat, the distorted reconstruction leads to a diagnosis that indicates sinus pause or sinoatrial block, which is a more serious problem. However, for the same pattern of error in the wireless channel, the perceived quality of the reconstructed signal degrades very gracefully in our framework, as shown in Figure 10(c), with the help of layered representation and by selectively recovering packets with higher priority.* This provides the physician with a better chance of arriving at an accurate diagnosis.*

## 6. Conclusions

In this paper, we proposed a layer-based representation of ECG data that classifies a group of samples into BL or one of the ELs according to their recorded sampling frequency, to achieve temporal scalability. The quality of ECG signal improves gradually as more ELs are appended to the BL. Because the samples of each layer are packetized separately, the loss of packets during transmission over a wireless channel does not always imply corruption of consecutive samples. We also proposed an efficient and simple real-time scheduling algorithm, the LB-EDF scheduling algorithm, for delivery of scalable ECG streaming data over a lossy wireless network to the medical staff for remote heart monitoring. It gives higher priority to the retransmission of BL and lower EL packets so that they have a greater chance of arriving at the RMS on time. This helps remote medical professionals to accurately read and interpret the ECG of a patient, even under severe channel conditions. The simulation result shows that, in terms of MSE, our approach outperforms the conventional scheme. The low complexity of the scheduling algorithm also makes it suitable for use in real-time ECG applications.

## Figures and Tables

**Algorithm 1:**
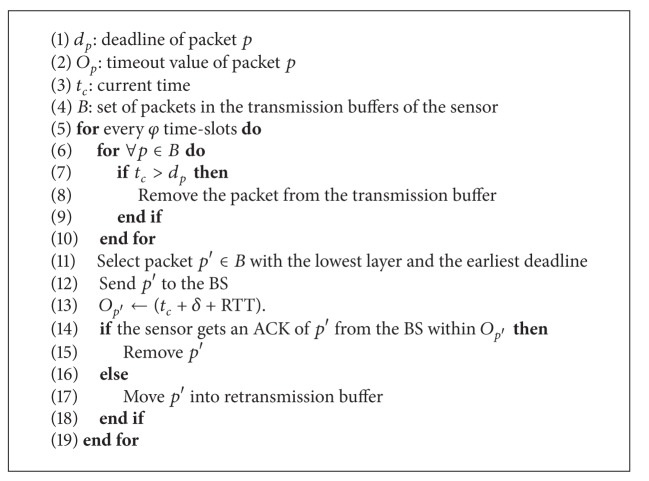
Layer-based EDF scheduling algorithm.

**Table 1:** Simulation parameters.

Symbol	Value(s)	Description
τ	1.67 ms	Duration of a time-slot
*φ*	3	Interlacing factor
*N*	3	Number of layers
*L*_pkt_	512 bits	Length of a packet
*L*_payload_	490 bits	Length of payload in each packet
*ϵ*	0 : 0.1	Steady-state PER
*v*	2 km/h : 5 km/h	Mobile speed of patients
*f*_*c*_	2.4 GHz	Carrier frequency
μ	307.2 kb/s	Reference channel data-rate
η	2	Number of leads
*R*	360 Hz	Samples per second
*L*_smpl_	11 bits	Sample size
μ_*s*_	3.96 kb/s	Data-rate of each ECG channel
μ_ecg_	7.92 kb/s	Total data-rate of ECG recordings
*δ*	1 time-slot	Feedback delay

**Table 2:** Markov parameters for sample PERs and mobile speeds.

*ϵ*	Mobile speed	*q*	*r*
0.1	2 km/h	0.006023	0.05420
	5 km/h	0.015012	0.13511

0.01	2 km/h	0.001849	0.18303
	5 km/h	0.004442	0.43980

0.001	2 km/h	0.000541	0.54067
	5 km/h	0.000872	0.87127

0.0001	2 km/h	0.000092	0.91553
	5 km/h	0.000099	0.98541
